# Barriers and facilitators to the recruitment of disabled people to clinical trials: a scoping review

**DOI:** 10.1186/s13063-023-07142-1

**Published:** 2023-03-08

**Authors:** Sameed Shariq, Alexandra M Cardoso Pinto, Shyam Sundar Budhathoki, Marie Miller, Suzie Cro

**Affiliations:** 1 School of Public Health, Imperial College London, London, UK; 2 Imperial Clinical Trials Unit, Imperial College London, London, UK

**Keywords:** Disability, Disabled people, Barriers and facilitators, Clinical trials, Inclusivity, Underrepresentation, Recruitment

## Abstract

**Introduction:**

Underrepresentation of disabled groups in clinical trials results in an inadequate evidence base for their clinical care, which drives health inequalities. This study aims to review and map the potential barriers and facilitators to the recruitment of disabled people in clinical trials to identify knowledge gaps and areas for further extensive research. The review addresses the question: ‘What are the barriers and facilitators to recruitment of disabled people to clinical trials?’.

**Methods:**

The Joanna Briggs Institute (JBI) Scoping review guidelines were followed to complete the current scoping review. MEDLINE and EMBASE databases were searched via Ovid. The literature search was guided by a combination of four key concepts from the research question: (1) disabled populations, (2) patient recruitment, (3) barriers and facilitators, and (4) clinical trials. Papers discussing barriers and facilitators of all types were included. Papers that did not have at least one disabled group as their population were excluded. Data on study characteristics and identified barriers and facilitators were extracted. Identified barriers and facilitators were then synthesised according to common themes.

**Results:**

The review included 56 eligible papers. The evidence on barriers and facilitators was largely sourced from Short Communications from Researcher Perspectives (*N* = 22) and Primary Quantitative Research (*N* = 17). Carer perspectives were rarely represented in articles. The most common disability types for the population of interest in the literature were neurological and psychiatric disabilities. A total of five emergent themes were determined across the barriers and facilitators. These were as follows: risk vs benefit assessment, design and management of recruitment protocol, balancing internal and external validity considerations, consent and ethics, and systemic factors.

**Conclusions:**

Both barriers and facilitators were often highly specific to disability type and context. Assumptions should be minimised, and study design should prioritise principles of co-design and be informed by a data-driven assessment of needs for the study population. Person-centred approaches to consent that empower disabled people to exercise their right to choose should be adopted in inclusive practice. Implementing these recommendations stands to improve inclusive practices in clinical trial research, serving to produce a well-rounded and comprehensive evidence base.

**Supplementary Information:**

The online version contains supplementary material available at 10.1186/s13063-023-07142-1.

## Introduction

Clinical trials hold a vital place in medical research, as the primary means by which the safety and effectiveness of a given medical, surgical, or behavioural intervention is learned. Clinical trials are conducted to approve a treatment for adoption into clinical practice and thus address the negative impact of disease on an affected population. However, disease affected populations are rarely homogeneous. Differences in impact and outcomes exist between groups across identifiers such as gender, ethnicity, socioeconomic status, and disability. The COVID-19 pandemic has drawn particular attention to this [1]. The causes behind these disparities in health are multi-faceted, but there is an acknowledgement that many of these stem from the research process that is foundational to modern medicine [2, 3].

Clinical trials often under-serve disadvantaged groups. This can happen as a result of one of three core problems: (i) under-representation of a group in a patient population of interest, (ii) a volume of research literature that is disproportionally lower than the prevalence of a specific condition, and (iii) a lack of research into the factors that influence differences in how groups are affected by interventions [4]. The under-serving of certain groups manifests in an inadequate evidence base on which to inform clinical management thus leading to suboptimal care and disparities in health.

Under-representation of minority groups in clinical trial research is a prevalent phenomenon, though who is affected is often context specific [4]. Making research more diverse is a priority of the Department of Health and Social Care (DHSC), as listed in their policy for future research [5]. The NIHR-INCLUDE project was commissioned in 2017 to serve this purpose and has since published the INCLUDE Ethnicity framework in 2020 [6]. This identified and addressed the barriers to meaningful inclusion for ethnic minorities in clinical research. However, many under-served groups remain that have no established frameworks for addressing their disproportionally low involvement, including people with disabilities. Moreover, it is essential that research institutions develop their own actionable frameworks to facilitate inclusion of under-served populations. Doing so will improve the generalisability of research, uncover non-physiological factors that drive differences in intervention uptake and outcomes, and serve to create more equitable healthcare [2].

People with disabilities are among those often under-represented and are considered one of the major under-served groups in medical research [4]. The World Health Organisation defines disability in the International Classification of Functioning, Disability and Health (ICF) as an umbrella term for impairments and limitations that have a significant and long-term negative effect on the ability to participate in activities [7]. Since the World Medical Association passed the Declaration of Helsinki in 1964, there has been an increased emphasis on the protection of vulnerable groups in research from the stresses and risks involved [8]. This was founded on the ethical principle that the wellbeing of human subjects should take precedence over interests of science and society. Unfortunately, this has often meant exclusion of people with disabilities rather than an implementation of measures that both accommodate their special needs and sufficiently include them. We define inclusion of people with disabilities in medical research to be where there is meaningful involvement and engagement with research activities to the same degree that this occurs for the rest of the study population. The first step to achieving this is the proportional recruitment and representation in study populations.

There will be many barriers and facilitators that influence the recruitment or lack thereof of disabled populations to clinical trials. Though literature reviews exist which explore one or multiple dimensions of these barriers and facilitators for populations with select disabilities [9, 10], there is yet to be an overarching summary of the evidence that considers the broader trends across disabled groups. Similarly, though there exist reviews that summarise in broad strokes the different factors that influence under-served groups at large [11], the evidence is insufficient to inform interventions for those living with impairments who, unlike other under-served groups, must navigate the impacts of a relative health limitation on their functioning as well as discriminatory treatment in society. We therefore chose to address the question, ‘What are the barriers and facilitators to recruitment of disabled people to clinical trials?’.

The primary aim of this study was to review published evidence and map ideas that exists on the barriers and facilitators to the recruitment of disabled people in clinical trials. We sought to identify knowledge gaps and ideas for further extensive research so that the root causes of under-representation of disabled people can be understood and addressed. The secondary aim was to identify insights from previous work that can help inform inclusive practice in future research.

## Methods

### Study design

**Fig. 1:**
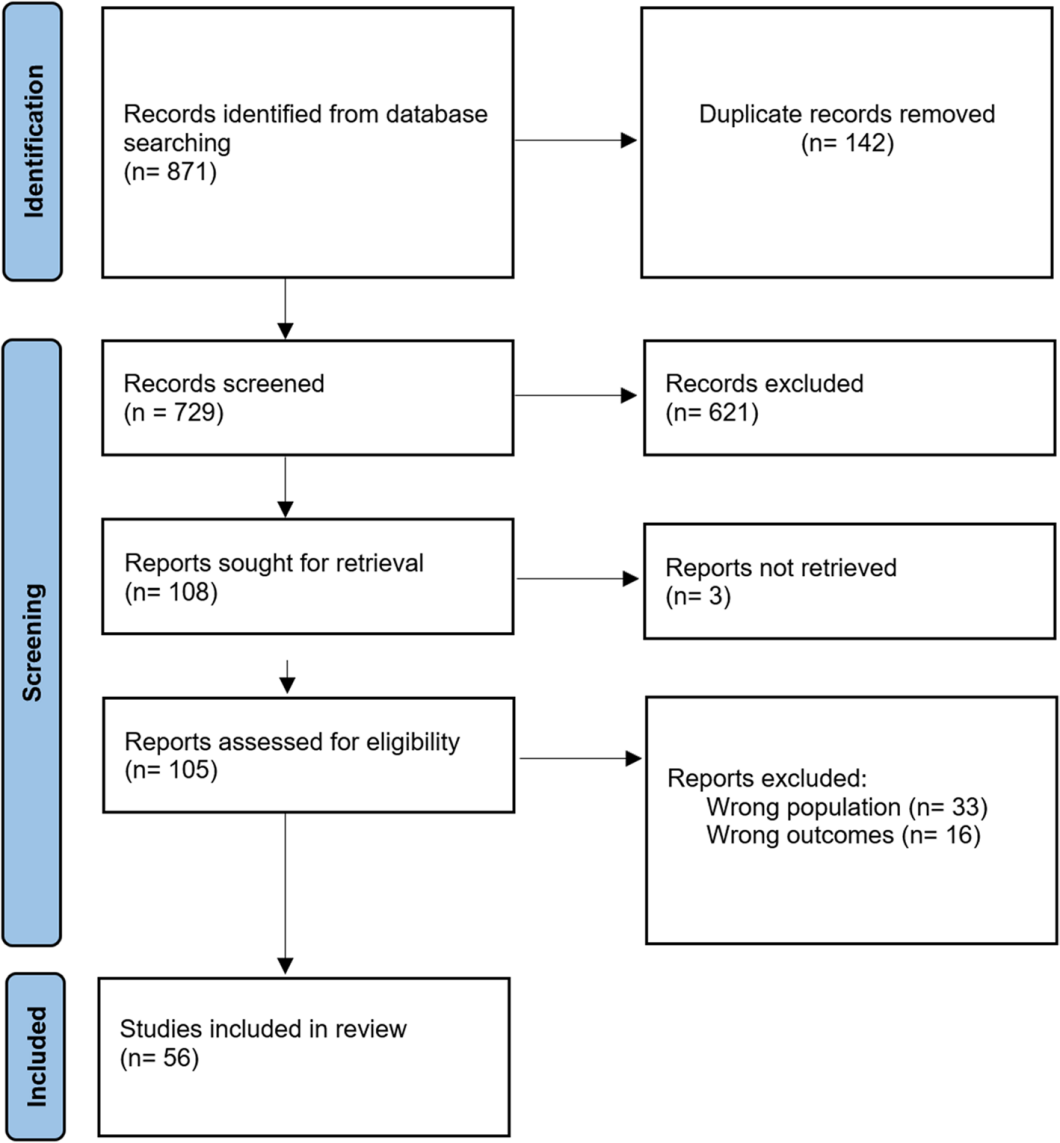
PRISMA flow diagram of study selection

A scoping review method was chosen as it allows us to explore the breadth of existing published literature, map and summarise the evidence, and identify any knowledge gaps that should be addressed by further research [12]. Preliminary literature searches revealed that evidence and ideas for barriers and facilitators exist in many different types of literature. Guidance was used from the Joanna Briggs Methods Manual for Scoping Reviews (JBI) [12]. The Preferred Reporting Items for Systematic Reviews and Meta-analysis extension for Scoping Reviews (PRISMA-ScR) was used and is reported in Additional File 3 (Fig. 1). The protocol for this review has not been registered.

### Data sources and search

The MEDLINE and EMBASE electronic databases were searched until 22 March 2022, with no date restrictions to collect as much relevant literature as possible. Only English language articles were included. The literature search terms were constructed with guidance from a college librarian over a course of three separate meetings. This was in accordance with the JBI Manual which recommends titles and key words of returned papers are analysed for relevance and then modified over an iterative process. The final search incorporated a combination of four key concepts from the research question: (1) disabled populations, (2) patient recruitment, (3) barriers and facilitators, and (4) clinical trials. Guidance on search terms for disabled populations set out by Walsh et al. was followed, as the search terms presented—developed in consultation with a multidisciplinary panel of experts in disability research—were deemed sufficiently sensitive and specific to the ICF definition of disability [13]. The full search strategy for both databases can be found in Additional File 1. The results of the literature search were imported into Covidence [14], which was used for screening by the first author.

### Study selection: eligibility criteria

The inclusion criteria required that papers discuss barriers or facilitators with reference to a recruitment in a clinical trials setting (Table 1). All barriers and facilitators, evidenced or hypothesised, were included so long as they made reference to the recruitment phase of the trial process. Any population that were described as having an ongoing disability that was covered by the ICF definition were included.

**Table 1:** The eligibility criteria used for study selection, organised by population, concept and context as per JBI Manual guidance

	Inclusion	Exclusion
Population	Populations defined for having a disability/disabilities covered by ICF definition. Any severity or presentation. Any age, sex, ethnicity, or income-level	Population groups who were not explicitly described for having a disability covered by ICF definition
Concept	Practical, ethical or theoretical factors identified as barriers or facilitators to recruitment	Barriers or facilitators relating to activities outside of research
	Hypothesised barriers or facilitators to recruitment identified by someone directly experiencing, caring for or researching disability	Barriers or facilitators to research inclusion in research processes apart from recruitment
	Strategies or interventions that were identified as being barriers or facilitators to recruitment	Factors having neither a clearly defined barrier nor facilitator effect on recruitment
Context	Clinical trials as a subject matter regardless of subtype	Medical research as a subject matter where clinical trials not explicitly mentioned
	Medical research as a subject matter where clinical trials explicitly mentioned	Healthcare or social care as a subject matter
	All study designs, time frames, and settings	

### Study selection: screening

The first author screened all papers for inclusion using Population, Concept, Context (PCC) information included in the eligibility criteria. Progress in study screening was discussed with the rest of the team in weekly-meetings over a 3-week period. This would involve the first author summarising the screening decisions over online video-call. Disagreements were discussed until consensus was reached and the eligibility criteria was refined to reflect these choices. Duplicates were removed automatically by the Covidence software.

### Data items and extraction process

Extracted data on study characteristics included country of origin, study design, participant type, type of disability, and type of evidence. Information about barriers and facilitators were extracted by the first author and an independent reviewer for a random selection of 10% of the papers (*n* = 6) and compared to identify any major oversights from the first author. Extraction from the rest of the papers was by the first author alone as no major oversights were noted throughout the independent verification. Extracted data was presented in weekly meetings over a 5-week period to a team from the Imperial Clinical Trials Unit to ensure clarity and communicability of ideas were maintained. All data was recorded in a data extraction form. Where there were issues in comprehension, data was extracted again and rediscussed. Risk of bias was not assessed for any of the papers, in accordance with the chosen scoping review methodology, as the purpose was to map the evidence .

### Data charting and synthesis

Barriers and facilitators were grouped thematically into key themes that emerged on review of the literature. Once established, these themes were reviewed by a second independent reviewer until no additional themes were identified. Themes were then grouped into a smaller list for ease and synthesised using tables and a narrative summary.

## Results

### Search results

A total of 729 studies were returned for screening following removal of duplicates. Following the abstract and title screening, 108 papers remained for full-text screening, which was again conducted by the first author. Thirty-three papers were excluded for having a population of focus that were not disabled under the ICF definition. This included populations at risk of having impairments but who were not confirmed to have one, such as at-risk populations eligible for Alzheimer’s screening, and homeless people among others. 16 papers did not discuss barriers or facilitators to recruitment. Three papers were not retrievable.

### Characteristics of included studies

A total of 56 eligible articles were included in this review. An overview of the characteristics of all included literature have been placed in Additional File 2.

The populations of interest for the included literature had many different disabilities which were broadly categorised. The most common disability types for the population of interest in the included studies were neurological (*N* = 24) and psychiatric (*N* = 14) disabilities. Physical, developmental, and sensory impairment disabilities were also included. Six studies looked at a combination of two or more types of disability. A full breakdown has been included in Table 2.

**Table 2:** Disabled groups included in this review

Type	Included disabilities	Number of articles (%)
Neurological	Dementia, stroke, multiple sclerosis, traumatic brain injury, cognitive impairment, Parkinson’s, neurofibromatosis	24
Psychiatric	Depression, psychosis, bipolar, addiction	14
Physical	Fibrodysplasia ossificans progressiva, frailty, mobility limitation, sarcopenia	6
Developmental	Intellectual disability, Down’s, Autism	5
Sensory impairment	Sensorineural hearing loss	1
Multiple/mixed	Type 1 diabetes + inherited retinal disease (as causes of visual impairment), frailty + trauma, persons lacking capacity to consent	6

**Fig. 2:**
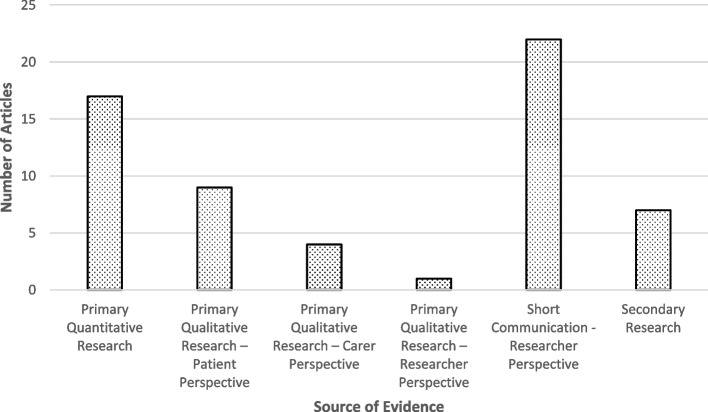
Types of evidence found in the review and the number of articles that drew on each of them. Types included were primary quantitative research (*N* = 17), primary qualitative research—patient perspective (*N* = 9), primary qualitative research—carer perspective (*N* = 4), primary qualitative research—researcher perspective (*N* = 1), short communication—researcher perspective (*N* = 22), and secondary research (*N* = 7)

Evidence about barriers and facilitators to clinical trials were sourced from articles drawing on Primary Quantitative Research on recruitment methods, Primary Qualitative Research with key stakeholders, and Short Communications by disability researchers. The most frequent source of evidence found in articles was Short Communications from Researcher Perspectives (*N* = 22) and Primary Quantitative Research (*N* = 17). A full breakdown has been included in Fig. 2. Further details about the type of evidence from each paper can be found in Additional File 2.

**Fig. 3:**
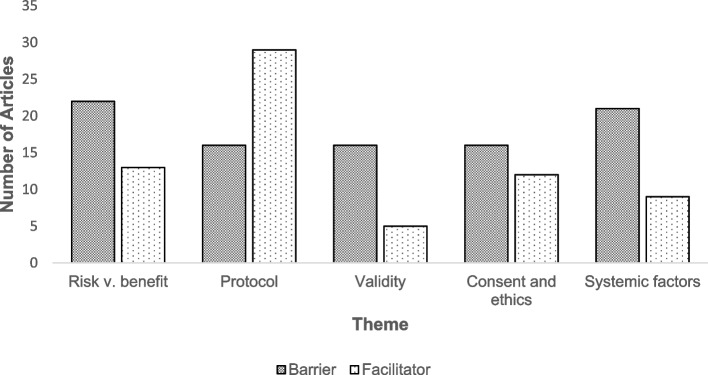
Barrier and facilitator themes found in the review and the number of articles from which that theme emerged. Risk vs benefit assessment was generated as a barrier by *N* = 22 and as a facilitator by *N* = 13. Protocol design and management was generated as a barrier by *N* = 16 and as a facilitator *N* = 29. Internal vs external validity was generated as a barrier by *N* = 16 and as a facilitator *N* = 5. Consent and ethics was generated as a barrier by *N* = 16 and as a facilitator *N* = 12. Systemic factors was generated as a barrier by *N* = 21 and as a facilitator *N* = 9

A total of five key themes were determined across the barriers and facilitators identified in this review. The most frequently mapped barrier themes were risks vs benefit assessments (*n* = 22) and systemic and logistical factors (*n* = 21). Facilitators were most frequently generated under the protocol design and management theme (*n* = 29). A full breakdown is included in Fig. 3.

#### Risks vs benefits assessment

The assessment of risk and benefit was found to be of particular concern for disabled people to clinical trial participation. Factors that increased the perceived risk to benefit ratio were barriers and included increased vulnerability to complications and side effects for certain disabilities [15–19], worries about unfair treatment impacting wellbeing [20–25], and low expectations of therapeutic benefit [26, 27]. Factors that decreased the perceived risk to benefit ratio were facilitators and included provisions of additional protections informed by a needs analysis [28] and open communication strategies to clarify and reassure [24, 27, 29]. This was generated as a barrier theme by 22 articles and as a facilitator theme by 13 articles (see Table 3).

**Table 3:** The barriers and facilitators mapped to the risk vs benefit assessment theme

Risk vs benefits assessment	
Barriers	**Facilitators**
**Safety** **risk** posed by trial interventions, practices. For example, medication washout was considered by both patients and health professionals to potentially worsen individual’s health experience of their disability during the trial As disabled people were **believed to be more vulnerable** to complications and side effects of invasive procedures, there was decreased willingness for recruitment [15–19]	Targeted **additional protections** informed by an **analysis of needs** was proposed by Hsiao et al. [28]. For example, people living with fibrodysplasia ossificans progressiva (FOP) were identified for being particularly vulnerable to repeated blood draw complications. To address this, Hsiao et al. suggests the procedures to be conducted by experienced phlebotomists with special directions
Disabled groups had increased perception of **risks to health, privacy and mental wellbeing** for taking part. For example, worries that recruitment would mean detachment from routine care that the person benefitted from [20], discomfort from trial procedures [21], confidentiality breaches [22], discrimination by staff [23], and placebo randomisation [24, 25]	Using **open communication** strategies that involved all stakeholders were found to be **reassuring** and successful at **clarifying** perceived threats [29]. This helped individuals to have realistic expectation and make informed decisions to enrol
Disabled groups had **low expectations of therapeutic benefit**. Due to disabled groups having often exhausted available medical options and research opportunities for resolving their impairment to little avail [26, 27]	**Detailed information** was found to be particularly appreciated by families and carers who showed preference for direct communication [24, 27]
Clinicians with long term contact with disabled patients **worried about loss of trust from patient and their family** [19]. Families and carers perceived a risk of **increased workload** if patient deteriorated from trial [21]	

#### Protocol design and management

This theme discussed the considerations pertaining to how clinical trial protocols were designed and managed. This would influence whether disabled participants were able and willing to be recruited. Barriers included having protocol designs that were missuited to accessibility requirements of disabled populations [21, 30] and the use of contact-based recruitment strategies which enabled discriminatory recruitment by staff [19, 31–33]. Facilitators included collaboration and co-design with disabled group representatives and other research bodies with experience in catering to additional needs [34–37]. Also, the use of innovative recruitment strategies to improve access to hard-to-reach populations. This was generated as a barrier theme by 16 articles and as a facilitator theme by 29 articles (see Table 4).

**Table 4:** The barriers and facilitators mapped to the protocol design and management theme

Protocol design and management	
Barriers	**Facilitators**
**Protocol design could be missuited to accessibility requirements**. For example, long study visits to research centre difficult for those with mobility limitations [21]. Virtual study visits proved harder for patients who had suffered traumatic brain injury (TBI) due to increased light sensitivity [30]	**Innovative recruitment schemes** like open access screening programmes, use of patient registries and community outreach programmes helps **improve access to hard-to-reach disabled populations** [28, 38–40]
Personal contact-based recruitment meant disabled groups were **less likely to be approached**. This was due to recruiters having **preconceived ideas that disabled people would be poor candidates** e.g. they would find the trial too stressful, dissent, be unable to participate, behave inappropriately, fail to adhere to treatment or create additional challenges for research staff [19, 31–33]	Cases of **collaboration and co-design** were reported to be positive for inclusivity. Examples include involving a **stakeholder coordinator** that had a personal connection to the research study’s disabled population of interest [34], involving **representatives from target populations** during question and protocol design [35], and **collaboration between research bodies** and trusted local groups working on similar goals. This helped build trust, allay concerns, allow knowledge sharing, and increase the number of referral pathways [36, 37]

#### Balancing internal vs external validity

Diversifying study populations is advantageous for generalisability of research but presents an extra challenge to the internal validity of a piece of research. Concerns around this balance influence researchers’ decision to recruit disabled groups. Barriers included the potential impact on safety and efficacy conclusions when including disabled participants [17, 41], low feasibility of internal validity measures such as medication washout [21, 27], the high prevalence of confounders in disabled groups [30, 42], and the use of unjustified and ambiguous research criteria by researchers [42]. Facilitators included the reporting of key baseline population characteristics [10] and the use of sensitive standardised assessment tools over clinical judgement [10, 33]. This was generated as a barrier theme by 16 articles and as a facilitator theme by 5 articles (see Table 5).

**Table 5:** The barriers and facilitators mapped to the balancing internal vs external validity theme

Balancing internal vs external validity	
Barriers	**Facilitators**
Certain disabilities may put participants at higher risk of **complications and side effects**, or make them less likely to have a meaningful **response to experimental therapy** which can impact **efficacy and safety conclusions** [17, 41]	**Reporting key baseline characteristics** for the study cohort and recruiting large sample sizes allows stratification of results and allows statistical adjustments to be carried out to account for covariates [10]
**Medication washout isn’t feasible** for many disabled people introducing risk of adverse drug interactions [21, 27]	Eligibility criteria can incorporate scores from **sensitive assessment tools** that relate to the confounding factor itself rather than proxies (e.g. using a Frailty Index instead of age) [10]
**Certain confounders are prevalent** among disabled populations and so exclusion from study populations greatly reduces generalisability [42]. For example, exclusion of people with psychiatric disorders from smoker populations [30]	Using **standardised tools rather than clinical judgement** may reduce the exclusion of individuals based on discriminatory biases that may be held by investigators [33]
Unjustified **ambiguous exclusion criteria** that leave exclusion largely to researchers discretion enables **discriminatory sampling** [42]	

#### Consent and ethics

Providing informed consent for clinical trial participation can be exclusionary or ethically problematic for disabled groups if appropriate considerations are not made. Barriers included reduced ability to provide informed consent from certain disabilities [34, 39, 41], gatekeeping by consent proxies [37, 43], and ethical restrictions barring direct contact between recruiters and institutionalised disabled groups [37]. Facilitators included accessible communication of consent information [27], involving disability group advocates [16, 43], and flexible capacity assessments [27]. This was generated as a barrier theme by 16 articles and as a facilitator theme by 12 articles (see Table 6).

**Table 6:** The barriers and facilitators mapped to the consent and ethics theme

Consent and ethics	
Barriers	**Facilitators**
Some disabilities may **directly limit** an individual’s ability to receive, comprehend, and use information about a clinical trial to provide **informed consent** [39]. Amongst these are sensory impairments, cognitive impairments, or communication difficulties [34, 41]	Where comprehension issues exist, **communicating information through more accessible means** has shown success. Examples include simplified and tailored consent forms, periodic quizzing of subjects, progressive disclosure strategies and trialled participation [27]
In cases where **proxies **are used, there is a risk of **gatekeeping **from carers, clinicians and institution managers [37, 43] resulting in the consent process being less of a reflection of patient priorities	**Easing** potential sources of **anxiety**, including patient’s **advocates**, and exploring patient **priorities** to reduce risk of coercion has been recommended [16, 43]
**Direct contact **between investigators and institutionalised disabled groups is restricted on the ethical grounds of **right to protection **however this can inadvertently deny them of their rights to hear about and get involved in research [37]	**Empowering patients to contribute to decision making** where possible when proxy consent is being used avoids gatekeeping. Having a sliding scale of required capacity for example, ensures that direct patient consent is considered as far as possible when the risk level is low enough to accommodate limited comprehension [27]
	**Formation of research committees to oversee involvement** of vulnerable institutionalised individuals who have fewer advocates will also protect right to inclusion [33]

#### Systemic factors

The systems and structures within which research is conducted can influence the ability and willingness of researchers to recruit disabled groups and disabled groups to enrol in research. Barriers included time and resource constraints on researchers making it difficult to pursue demanding inclusive practices [9] and a lack of support and guidance from research bodies on inclusive practices [33, 41]. Facilitators included greater involvement and advocacy for inclusive practice by ethics review boards [10] and additional resource provision by research bodies to researchers [40, 44]. This was generated as a barrier theme by 21 articles and as a facilitator theme by 9 articles (see Table 7).

**Table 7:** The barriers and facilitators mapped to the systemic factors theme

Systemic factors	
Barriers	**Facilitators**
**Inclusive practices** are often more **burdensome** for researchers whose work is already **time and resource constrained** [9]. This is because the provision of things like additional protection measures and widening recruitment strategies are time-consuming and resource-demanding	**Ethics review boards and editors** can implement requirements for researchers to **report on the sociodemographic** compositions of their sample as well as **justify their protocol design choices according to inclusive criteria** [10]
Researchers feel **unsupported and unsure** as to whether their inclusive approaches are sufficiently effective and ethical [33, 41]	**Local research agencies** can also help equip researchers with **equipment and resources** they need to conduct their research in non-traditional settings [35]. This may extend to sophisticated screening tools and recruitment registries that streamline **recruitment to be widened and more sensitive** so as many people with a given disability can be found as possible [40, 44]

## Discussion

This review has collected evidence in five major areas for consideration when strategizing for representation of disabled people in clinical trials. The first is understanding and accommodating factors that increase the risk to benefit ratio of enrolment. The second is exploring protocol design and methods that maximise accessibility for disabled groups. The third is addressing the challenge to internal validity posed by diversifying study populations. The fourth is practicing consent processes that balance ethical inclusion with the right to protection of disabled groups. The fifth is enabling inclusive practice in research on a larger institutional level. This information will help researchers develop and refine evidence-based strategies that target established barriers to participation for disabled people.

Reconciling the risks involved in clinical trial recruitment was found to be a particular barrier for disabled groups. The findings of this review, which point to the increased vulnerability of disabled people both physically and socially as barriers to recruitment, are supported by wider literature. Disabled groups have faced historical discrimination which persists to this day [45], and the detrimental impact this has on taking risks, such as those posed by trial enrolment, is made clear by protection motivation theory [46]. Future work must therefore explore strategies that reduce these risks for disabled people and improve their coping capacity. The review highlights key challenges that have been faced in this endeavour, such as tailoring these approaches to each disabled group’s specific needs. Currently, there is evidence that shows increased trial participation when study teams use a data driven approach that offers insights into population needs and priorities [47]. Future research should be mindful of this and take care not to make assumptions where barriers are highly context-specific.

The review also revealed there are considerable influences on patient enrolment outside of the patient and researcher dyad, including from family, paid carers, and clinicians. However, there is a gap in the literature with very few articles identified as reporting these perspectives. Future research should explore this as there is typically a high degree of dependency to these stakeholders from the patient. Considerations of personal consequences, capacity, and logistical coordination from these parties were found to have determining influences on disabled patient recruitment to trials, due to the high dependency relationships with disabled individuals. Despite this, the number of articles that drew on their perspectives was relatively low illustrating a key gap in the literature that future research must address.

Another key finding of the review was that many barriers stemmed from a disconnect between researcher expectations and disabled patients’ realities. Of vital necessity, therefore, is the utilisation of co-design and community consultation when designing protocols. This will ensure that the needs of each potential study cohort are accommodated. Though the wider literature has shown there are challenges to these collaborative partnerships, such as maintaining methodological rigor and striking balances in power dynamics [48], there are also significant benefits including better access to participants and greater likelihoods of engagement [49, 50]. Incorporating these features into future clinical trials, particularly those aimed specifically at disabled groups, also serves an ethical “emancipatory” purpose founded in the social model of disability as there is an active challenge of dominant hierarchies of knowledge which have in part perpetuated historical under-serving of disabled people [51]. Taking these practices further therefore will tackle the broader issue of under-serving both on a practical and ideological level.

The review has identified key reasons why those with disabilities would face extra hurdles in providing informed and sound consent and possible means to maximise patient-directed consent.

Person-centred approaches, as described in this review and which include tailoring of consent processes, the use of decision aids to improve accessibility, and revisiting consent after data collection, have been regarded as successful to improving inclusion for disabled groups in wider literature [52, 53]. However, such approaches are likely to increase demands on researchers who wish to uphold the methodological rigor of their clinical trials. These approaches often necessitate longer and more involved periods of contact with study participants as well as extra resources to meet complex needs [54]. Novel strategies will need to be explored and adopted to overcome added challenges of high attrition or fluctuating levels of capacity and adherence [55]. Though these approaches have begun being explored [56], further research and evidence on their potential benefit is warranted.

The key ethical issue of balancing a right to inclusion and a right to protection also lies at the heart of consent considerations. The United Nations Convention on the Rights of Persons with Disabilities, adopted in 2006, broached the issue by calling for a change to viewing and treating disabled people as “objects” of social protection and instead as “subjects” with rights to make decisions [57]. The most recent revision of the World Medical Association’s Helsinki declaration permitted involvement of adults without the capacity to consent in research without direct benefit even if future patients could benefit [8]. This acknowledgement that exclusion from research is ultimately detrimental to the purpose of protecting disabled groups is growing in research ethics [58]. It is therefore important to rework overbearing protection measures so that they do not hamper attempts to address accessibility barriers such as those described in this review.

The review has mapped some key ways in which internal validity considerations have disincentivised the recruitment of disabled groups, including concerns about study power due to confounders. Wider literature shows this to be a common concern where the wider target populations are heterogeneous and particularly when there is treatment effect heterogeneity among them [59, 60]. Though the review highlights key ways in which internal and external validity can be better balanced and provides direction for more inclusive and effective eligibility criteria implementation, it may be the case that prioritisation of internal validity over external validity and vice versa will vary depending on trial design and phase. Pragmatic trials, for example, are where interventions are tested in real life settings to evaluate effectiveness in routine practice comparative to existing interventions to inform clinical decisions in practise [61], whereas explanatory trials seek to confirm a clinical efficacy hypothesis under ideal conditions [62]. Pragmatic trials are typically conducted to supplement the knowledge generated in traditional explanatory clinical trials, as pragmatic trials will be unable to maintain internal validity to the same degree by virtue of their higher variance in their patient population [63]. It may be argued then that the aim to improve diversity and inclusivity to trial research should be targeted to later phase trials. Ultimately, evidence from trials must, in summation, do justice to the potential of an intervention while also accurately establishing their generalisability in order to serve all groups.

Development of infrastructure within academic, philanthropic, and government institutions has been identified as a core action necessary to improve inclusion within this review and in wider literature [64]. There are examples of research groups developing guidance for research on specific areas of disability, such as IMPACT’s guidelines on Traumatic Brain Injury research [65]. However, dedicated bodies that researchers are obliged to follow must promote inclusivity as well. In the UK, the Research Governance Framework asks researchers to take account of disability where relevant [66]. The INCLUDE project has produced guidance on how to promote inclusivity throughout the research process [4]. Pushes from national scale bodies like this enable a culture of collaboration which have been key for cross-sector achievements like the integration of screening services and recruitment registries into primary care [67]. Institutions, editors, and review boards in direct contact with researchers must ensure such guidance is implemented as well so that inclusive approaches and monitoring become standard practice.

### Strengths and limitations

To our knowledge, this is the first wide reaching summary that did not focus on a single dimension of barriers and facilitators or on particular disabled groups. The only previous review to synthesise barriers and facilitators to trial inclusion did not appreciate the additional complexities of serving disabled populations in a clinical trial setting [11]. An evidence-based search strategy was employed to include all disabilities covered by the ICF definition. As a result, the distribution of articles across type of disability can be used to identify gaps in the literature.

Nevertheless, this study has limitations that should be acknowledged. This review did not engage active Patient and Public Involvement (PPI) in key areas where it may have benefitted the study, such as refinement of the search strategy and the data extraction process. Documented barriers and facilitators were not always substantiated with robust quantitative or qualitative evidence due to the inclusion of commentary and opinion pieces which posited ideas based on single or limited perspectives, albeit sufficiently relevant ones. The data was also not formally appraised for quality as scoping reviews are a method of mapping the available evidence, independent of quality, instead. Therefore, further research will be needed to assess and establish the impact of these proposed barriers and facilitators.

Tackling under-representation of disabled people in clinical trials requires high retention alongside adequate recruitment, but there is evidence to show this varies significantly for disabled groups [68]. Though there will be significant overlap, investigation of trial retention strategies must also be performed in the future, which was omitted by this review. Indeed, inclusivity will need to be improved throughout the research process. Factors such as age, socioeconomic status, and ethnicity can interact with disability to influence people’s experience [69, 70]. For example, their level of care access or advocacy capacity which, in turn, will have consequences on their enrolment to trials. Recognising the importance of intersectionality, we have recorded additional sociodemographic characteristics for the populations addressed in this study beyond their disability type in Additional File 2. However, we did not incorporate intersectionality into our mapping of the evidence. This was because of the wide variation in study types of the included literature owing to the broad scope of the review. There was also insufficient sociodemographic stratification of given barriers and facilitators in the included literature. Future reviews that have a narrower scope should prioritise an intersectional approach where possible.

## Conclusions

This study delivers key insights on barriers and facilitators to the inclusion of disabled people in clinical trials to better inform inclusive practices and provide direction to future inclusivity research for disabled people. Appreciating that accessibility requirements are unique to each disabled group and their context and embedding principles of co-design into research protocols is a key strategy to improving recruitment. Empowering disabled people to exercise their right to choose and right to inclusion while upholding their right to safety, for example, with person-centred approaches to consent, should be adopted. Empowerment must go both ways, however, to researchers as well as study populations. Research bodies and institutions must therefore play an active role in supporting and promoting inclusive practices with resources, guidance, and regulation. Future research on inclusivity should build on these broad foundational approaches to deliver specific insights for research practice. Future research should prioritise these approaches and aim to generate a well-rounded evidence base that serves disabled groups with justice.

## Supplementary Information

**Additional file 1.** Detailed review search strategy. Full search strategy applied used to search MEDLINE and EMBASE via Ovid.

**Additional file 2.** Characteristics of included studies. Full table showing study characteristics of each paper included in this review.

**Additional file 3.** PRISMA Extension for Scoping Reviews Completed Fillable Checklist.

## Data Availability

The datasets used and/or analysed during the current study are available from the corresponding author on reasonable request.

## Abbreviations

ICF: International Classification of Functioning, Disability and Health
JBI: Joanna Briggs Methods Manual for Scoping Reviews
PRISMA-ScR: Preferred Reporting Items for Systematic Reviews and Meta-analysis extension for Scoping Reviews
