# Supplementary material for: Barriers and facilitators to the recruitment of disabled people to clinical trials: a scoping review
Source: Trials. 2023 Mar 8;24:171. doi: 10.1186/s13063-023-07142-1 (PMC9994780; doi:10.1186/s13063-023-07142-1)
Supplement: Supplementary file 1 — Additional file 1. Detailed review search strategy. Full search strategy applied used to search MEDLINE and EMBASE via Ovid. [file 13063_2023_7142_MOESM1_ESM.docx]

**Additional File 1**: Detailed review search strategy

MEDLINE and EMBASE via Ovid

1 ("Disabled person*" or "disabled people" or disabilit* or "hearing impair*" or amputee* or "mentally ill" or "mentally disabled" or "mental* retard*" or "mobility limitation*" or "developmental disab*" or "dependent ambulation" or quadriplegi* or paraplegi* or hearing loss or blindness or "assistive technology" or "vision disorder*" or "mental disorder*" or "psychiatric disab*" or handicapped or "functional limitation*" or "activity limitation*" or "mobility impair*" or "vision impair*" or "hearing impair*" or "cognitive impair*" or "intellectual disabilit*" or "participation limitation*").mp. [mp=title, abstract, original title, name of substance word, subject heading word, floating sub-heading word, keyword heading word, organism supplementary concept word, protocol supplementary concept word, rare disease supplementary concept word, unique identifier, synonyms]

2 (challenge* or needs or barrier* or issue* or difficult* or obstacle* or problem* or motivator* or facilitat* or overcome* or help*).mp. [mp=title, abstract, original title, name of substance word, subject heading word, floating sub-heading word, keyword heading word, organism supplementary concept word, protocol supplementary concept word, rare disease supplementary concept word, unique identifier, synonyms]

3 ("research participation" or "research inclusi*" or underrepresentation or underserv* or "patient recruitment" or "patient selection" or "patient retention" or "patient enrolment" or "study recruitment" or "study selection" or "study retention" or "study enrol*ment" or "trial recruitment" or "trial selection" or "trial retention" or "trial recruitment" or "trial selection" or "trial retention" or "trial enrolment").mp. [mp=title, abstract, original title, name of substance word, subject heading word, floating sub-heading word, keyword heading word, organism supplementary concept word, protocol supplementary concept word, rare disease supplementary concept word, unique identifier, synonyms]

4 Clinical Trials as Topic/

5 1 and 2 and 3 and 4

6 Patient Selection/

7 Disability Studies/

8 disabled persons.af.

9 Disabled Persons/

10 3 or 6

11 1 or 7 or 9

12 2 and 4 and 10 and 11

13 (clinical trial* or treatment trial*).mp. [mp=title, abstract, original title, name of substance word, subject heading word, floating sub-heading word, keyword heading word, organism supplementary concept word, protocol supplementary concept word, rare disease supplementary concept word, unique identifier, synonyms]

14 4 or 13

15 2 and 10 and 11 and 14
