# Supplementary material for: Barriers and facilitators to the recruitment of disabled people to clinical trials: a scoping review
Source: Trials. 2023 Mar 8;24:171. doi: 10.1186/s13063-023-07142-1 (PMC9994780; doi:10.1186/s13063-023-07142-1)
Supplement: Supplementary file 2 — Additional file 2. Characteristics of included studies. Full table showing study characteristics of each paper included in this review. [file 13063_2023_7142_MOESM2_ESM.docx]

| Ref No | Item | Country of origin | Study Design | Evidence Source | Disabled Population of Focus | Additional Reported Characteristics of Disabled Population of Focus | Results: Barriers | Results: Facilitators |
| --- | --- | --- | --- | --- | --- | --- | --- | --- |
| (1) | Brooks (2020) | Canada | Qualitative Study | Primary Qualitative Research – Carer Perspective: Qualitative interview study with parents of children living with Type 1 Diabetes (T1D) (n=15) and parents of children living with Inherited Retinal Diseases (IRD) (n=14) | Type 1 Diabetes or Inherited Retinal Diseases (as causes of Visual Impairment) | Age: All participants were under 18. All disabled children’s ages recorded at time of diagnosis and at time of interview.  Residence: All participants live in Canada. Canadian province of residence for each family was also given.  Family History: 22/29 had no known family history of T1D or IRD. Whether there was known prior family history of T1D or IRD was recorded. | Risks vs benefit assessment  Systemic and logistical factors | Risks vs benefit assessment  Protocol design and management |
| (2) | Witham (2018) | UK | Commentary/Opinion/Editorial | Short Communication - Researcher Perspective: Opinion piece on how to improve Sarcopenia clinical trials by experienced Sarcopenia researcher with reference to wider literature. | Sarcopenia | Age: Author includes discussion on developing capacity for running sarcopenia trials in elderly (>65) populations specifically. | Systemic and logistical factors | Protocol design and management  Systemic and logistical factors |
| (3) | Park (2019) | USA | Case Report / Series | Primary Quantitative Research: Case report of a successful implementation of a recruitment strategy for early phase clinical trials in people with MCI and AD. | Mild Cognitive Impairment (MCI) and Alzheimer’s Disease (AD) | Age: Mean age reported for people with AD was 77 years, and for people with MCI was 71 years. | Risks vs benefit assessment | Risks vs benefit assessment  Protocol design and management |
| (4) | Bayer (2003) | UK | Commentary/Opinion/Editorial | Short Communication - Researcher Perspective: Opinion piece on the ethical rationale for adopting more inclusive practices with reference to wider literature. | Mild Cognitive Impairment | Age: Author’s discussion primarily revolves around age-related cognitive impairments in the elderly (>65).  Institutional status: Author includes discussion on specific challenges with institutionalised individuals with cognitive impairment. | Risks vs benefit assessment  Protocol design and management  Internal vs external validity  Consent and ethics  Systemic and logistical factors | Risks vs benefit assessment  Consent and ethics |
| (5) | Humphreys (2015) | USA | Systematic review | Secondary Research: Systematic review of highly cited randomised control trials with specific emphasis on the employment of eligibility criteria that excluded people with a psychiatric disorder. | Psychiatric disorders | N/A | Internal vs external validity  Systemic and logistical factors | Consent and ethics |
| (6) | Fisher (2002) | USA | Commentary/Opinion/Editorial | Short Communication - Researcher Perspective: Opinion piece on ethical issues surrounding the inclusion of suicidal individuals into controlled settings of clinical trials. | Suicidal depression | N/A | Internal vs external validity  Protocol design and management  Consent and ethics | Protocol design and management  Consent and ethics |
| (7) | Carey (2001) | USA | Qualitative Study | Primary Qualitative Research – Patient Perspective: Qualitative interviews with outpatients of mental health clinic living with severe and persistent mental illness (SPMI) (n=45) | Severe and Persistent Mental Illness | Gender: Participants were evenly split between male (n=22) and female (n=23).  Ethnicity: Backgrounds included European-American (67%), African American (27%), Native American (5%), and others (2%)  Disability sub-type: Mental illnesses of cohort included schizophrenia (31%), schizoaffective disorder (13%), major depression (35%), bipolar disorder (15%). |  | Risks vs benefit assessment  Protocol design and management |
| (8) | Jensen (2019) | New Zealand | Literature Review | Secondary Research: Literature review of geriatric trauma trials with focus on methodological aspects that hindered inclusion. | Elderly with trauma | N/A | Internal vs external validity  Systemic and logistical factors  Consent and ethics | Protocol design and management  Consent and ethics |
| (9) | Trivedi (2015) | USA | Literature Review | Secondary Research: Narrative literature review of eligibility criteria in studies focusing on treatment for neurological disorders. | Neurological disorders | N/A | Internal vs external validity | Protocol design and management  Systemic and logistical factors  Internal vs external validity |
| (10) | Schneider (1997) | USA | Case Report / Series | Primary Quantitative Research: Application of typical clinical trial eligibility criteria to assess exclusion impact on sample population of Alzheimer’s patients. | Alzheimer's disease | Age: Provisionally ineligible participants had a mean age of 76.3 years and provisionally eligible participants had a mean age of 74.8 years  Ethnicity: Provisionally ineligible participants were 78% white, 8% black, and 10% hispanic while provisionally eligible participants were 84% white, 5% black, and 6% Hispanic.  Education: 36% of provisionally ineligible participants had a college education while 46% of provisionally eligible participants had a college education.  Income: Median yearly income range for provisionally ineligible participants was $10,000-$14,999 while for provisionally eligible participants it was $20,000-$24,999 | Internal vs external validity |  |
| (11) | Lehoux (2005) | USA | Qualitative Study | Primary Qualitative Research – Patient Perspective:  Qualitative interviews with recent-onset psychosis patients (n=25) who refused to participate in a clinical trial on psychosis which utilised an acquaintanceship recruitment procedure. | Psychosis patients | Disability sub-type: Disorder categorisation of psychosis was recorded including schizophrenia (N = 16), schizoaffective disorder (N = 4), delusional disorder (N = 2), schizophreniform disorder (N = 2), and psychosis not otherwise specified (N = 1) | Risks vs benefit assessment |  |
| (12) | Karlawish (2001) | USA | Commentary/Opinion/Editorial | Short Communication - Researcher Perspective: Opinion piece focusing on ethical issues surrounding the inclusion of Alzheimer’s patients into clinical trials. | Alzheimer's/Dementia | N/A | Risks vs benefit assessment  Consent and ethics | Consent and ethics |
| (13) | Humphreys (2000) | USA | Quantitative study | Primary Quantitative Research: Application of typical clinical trial eligibility criteria to assess exclusion impact on sample population of people living with psychiatric disorders. | Psychiatric disorders | Gender: Men (N=391) composed slightly under two-thirds of the sample (65.9%).  Marital status: At intake, 211 (35.6%) participants were separated or divorced, and 167 (28.2%) were married or living in a marriage-like relationship.  Employment status: About two-thirds of the sample (N=380, 64.1%) were unemployed.  Ethnicity: The most common racial/ethnic backgrounds were non-Hispanic Caucasian (51.9%, N=308) and African American (34.2%, N=203).  Age: The mean age of the participants was 39.0 years (SD=10.5) | Internal vs external validity |  |
| (14) | Hammers (2019) | Switzerland | Quantitative Study | Primary Quantitative Research:  Statistical analysis of data on characteristics of people who had passed and failed AD screening for recruitment. | Alzheimer's disease | Age: The mean age was 72.5 years old (+/− 7.1 years)  Education: The mean time spent in education was 16.4 years |  | Protocol design and management |
| (15) | Jones (2019) | UK | Commentary/Opinion/Editorial | Short Communication - Researcher Perspective: Editorial short piece describing the documented barriers to inclusion of mental health patients in clinical trials found in their journal with emphasis on researcher experiences of navigating these. | Mental health disorders | N/A | Protocol design and management  Consent and ethics  Systemic and logistical factors | Protocol design and management |
| (16) | Heller (2006) | USA | Commentary/Opinion/Editorial | Short Communication - Researcher Perspective: Article summarising the history of approaches to researching children with Down Syndrome and discusses which research principles to prioritise. | Down Syndrome | N/A | Protocol design and management  Internal vs external validity  Systemic and logistical factors |  |
| (17) | McQueen (2008) | USA | Qualitative Study | Primary Qualitative Research – Patient Perspective and Carer Perspective: Qualitative surveys with adult patients or parents of child patients with Neurofibromatosis Type 1 (n=74) and care providers of these patients (n=69) | Neurofibromatosis | Age: Survey asked for age of relevant patient to be stated. Median age was 34.5 years old.  Gender: Survey Q asking individual to identify gender as either male or female. The majority of patient respondents (57%) were women.  Family History: Whether there was a family history of Neurofibromatosis was recorded. 41% noted a family history of NF1. |  |  |
| (18) | Lembke (2016) | UK | Commentary/Opinion/Editorial | Short Communication - Researcher Perspective: Commentary piece on the reasons for greater inclusion of people with mental illness in smoking cessation research. | Mental illness and addiction | Smoking status: Discussion centres around intersection between active smokers and people living with mental illness. | Internal vs external validity  Systemic and logistical factors | Risks vs benefit assessment  Systemic and logistical factors |
| (19) | Myers (2020) | Netherlands | Quantitative Study | Primary Quantitative Research: Paper describing the results of a recruitment drive for a study on Parkinson’s functionality and descriptive statistics on reasons for and against enrolment. | Parkinsons Disease | Ethnicity: Participants who were from Ashkenazi Jewish descent made up 76% of cohort (linked to genetic trait of interest in study)  Age: Mean age of recruited cohort was 56 years old  Gender: 59% of recruited cohort were female |  | Protocol design and management |
| (20) | Fox (2017) | UK | Commentary/Opinion/Editorial | Short Communication - Researcher Perspective: Commentary piece on how to change clinical trial designs in ways that will enable more accurate and inclusive recruitment of people with MS. | Multiple Sclerosis | N/A |  | Internal vs external validity |
| (21) | Diver (2017) | Netherlands | Qualitative Study | Primary Qualitative Research – Patient Perspective and Carer Perspective:  Qualitative semi-structured interview study with stroke survivors (n=11) and carers of stroke survivors (n=5) | Stroke | None given. | Risks vs benefit assessment  Consent and ethics |  |
| (22) | Shapiro (2017) | USA | Case Report / Series | Primary Qualitative Research – Patient Perspective: Questionnaire survey study with African-American church-goers in Atlanta with declared disability (n=221) about various influences on clinical trial participation | All | Ethnicity: All participants were African-American  Age: Mean age of participants was 63.6 years  Gender: 78% of cohort were recorded as female  Education level: 64% of participants had an associate’s degree or higher  Income: Moderately spread with 66.1% having less than $60,000  Marital status: Participants were primarily married (n=102, 46.2%) or separated/divorced (n=59, 26.7%) | Systemic and logistical factors | Consent and ethics  Systemic and logistical factors |
| (23) | Helmchen (1981) | USA | Commentary/Opinion/Editorial | Short Communication - Researcher Perspective: Author reflects on how to establish valid consent for people with mental illness and how this shapes recruitment. | Psychiatric disorders | Disability Sub-type: Author reflects on how different severities of psychiatric impairment will lead to different requirements and outcomes. | Risks vs benefit assessment  Consent and ethics  Protocol design and management |  |
| (24) | Rosende-Roca (2021) | Spain | Quantitative Study | Primary Quantitative Research: Statistical analysis of data on cohort to whom AD screening criteria was applied. | Alzheimer's | Gender: The cohort comprised of 2926 men (30.5%) and 6667 women (69.5%). | Internal vs external validity |  |
| (25) | Wojcik (2019) | Poland | Case Report / Series | Primary Quantitative Research: Statistical analysis of effectiveness of open-access screening program as a clinical trial recruitment strategy | Alzheimer's | N/A | Protocol design and management |  |
| (26) | Studenski (2009) | France | Commentary/Opinion/Editorial | Short Communication - Researcher Perspective: Commentary piece covering what is known about different  factors that can be used for the selection of a target population,  current gaps in knowledge and priorities for future Sarcopenia research. | Sarcopenia | N/A |  | Protocol design and management |
| (27) | Witham (2020) | UK | Commentary/Opinion/Editorial | Short Communication - Researcher Perspective: Guideline document on broad principles to apply in order to improve inclusivity of COVID-19 research for underserved groups including disabled | All | N/A |  | Protocol design and management  Systemic and logistical factors |
| (28) | Withall (2020) | UK | Case Report / Series | Primary Quantitative Research:  Cost and effectiveness analysis of different invitation methods as trial recruitment strategy for people with mobility limitations. | Mobility disability patients | N/A |  | Protocol design and management  Systemic and logistical factors |
| (29) | Kannisto (2017) | Canada | Quantitative Study | Primary Quantitative Research: Recruitment and follow-up processes of a previous clinical trial were monitored and analyzed to provide picture of dropout predictors | Serious Mental Illness | Age: eligible group tended to be significantly younger (mean 39.2, SD 13.2 years, P<.001) than those who were not eligible (age: mean 43.7, SD 14.6 years).  Gender: Eligible group tended to be more often women (2103/4181, 50.30%) than those who were not eligible (women: 3633/6514, 55.78%)  Marital Status: Whether individual was married or not was recorded but presented as an association statistic with risk of dropping out.  Employment Status: The employment status was recorded was recorded but presented as an association statistic with risk of dropping out.  Education: Whether the individual had a vocational education was recorded was recorded but presented as an association statistic with risk of dropping out. | Risks vs benefit assessment |  |
| (30) | Emre (2014) | USA | Commentary/Opinion/Editorial | Short Communication - Researcher Perspective: Commentary piece on current way of managing disability and practical challenges to conducting clinical trials including recruitment | Parkinson's and Dementia/Cognitive Impairment | N/A | Consent and ethics | Consent and ethics |
| (31) | Rookhuijzen (2014) | Netherlands | Qualitative study | Primary Qualitative Research – Patient Perspective: Qualitative observation and a standardized interview with the older person and a close relative who could act as a proxy (surrogate) decision maker, if necessary (n=18) | Cognitive Impairment | Gender: Whether participants identified as male or female was recorded. 67% of cohort was female.  Age: The age of participants was recorded. All people were over 75 years old, mean age was 79.9 years.  Marital status: Whether the participant was currently married or not was recorded. 56% were married. |  |  |
| (32) | Kogan (2009) | UK | Quantitative Study | Primary Quantitative Research: Statistical analysis of results from application of a new recruitment method developed to enhance the ethnic/racial and  socioeconomic diversity of participants in Bipolar Disorder trials. Community sites were trialled for recruitment to see if there was greater diversity as compared to recruitment at academic sites. | Bipolar Disorder | Age: Community site participants were generally older (42.8 years vs 38.8 years  Gender: Community site participants were more likely to be females  (70.3% vs. 60.3%)  participants.  Education: Participants from academic sites had  higher rates of post high school education (college  degree: 28.1% vs. 13.6%; postgraduate:  20.2% vs. 4.5%,  Employment status: Community  participants having higher rates of unemployment  (29.6% vs. 21.2%) | Protocol design and management  Internal vs external validity  Systemic and logistical factors | Protocol design and management |
| (33) | Veenstra (2010) | USA | Qualitative Study | Primary Qualitative Research – Researcher Perspective: Qualitative semi-structured interviews with researchers with experience in trials for people with Intellectual Disabilities (n=18) | Intellectual Disability | None given. | Protocol design and management  Systemic and logistical factors |  |
| (34) | Africano-Oliver (2010) | UK | Case Report | Short Communication - Researcher Perspective: Commentary piece on recruitment challenges faced by researchers in clinical trial for people with intellectual disability | Intellectual Disability | N/A | Protocol design and management  Risks vs benefit assessment | Protocol design and management  Consent and ethics |
| (35) | House (2020) | USA | Case Report / Series | Short Communication - Researcher Perspective: Case study discussion of individual participants and challenges surrounding choice to recruit into clinical trial for people with cognitive impairment. | Severe cognitive impairment | N/A | Risks vs benefit assessment  Internal vs external validity  Consent and ethics | Protocol design and management  Consent and ethics |
| (36) | Grill (2010) | UK | Literature Review | Secondary Research: Literature review on AD recruitment and retention with focus on prominent barriers and challenges currently in literature. | Alzheimer's | N/A | Protocol design and management  Risks vs benefit assessment  Systemic and logistical factors | Protocol design and management |
| (37) | Ridda (2008) | Australia | Case Report / Series | Short Communication – Researcher Perspective: Paper discusses recruitment process for a clinical trial which successfully enrolled 315 frail elderly to reveal strategies for success. | Frailty | Gender: 147 women and 168 men were recruited.  Age: The cohort mean age was 70, median age was 66.7 and the age ranged from 60 to 102 years.  Mental Capacity: Thirty-four of 315 (10.8%) had a MMSE score of less than 20, therefore required a guardian consent; out of those 34 patients, four were completely not assessable for the MMSE. | Risks vs benefit assessment  Systemic and logistical factors  Internal vs external validity | Protocol design and management |
| (38) | Jefferson (2008) | USA | Quantitative Study | Primary Quantitative Research: Statistical analysis of neuropsychological functioning markers between cognitively normal population (n=40) and a population with Mild Cognitive Impairment (n=40) to determine factors around capacity to provide informed consent. | Mild Cognitive Impairment | Age: Mean age of MCI population was 74.3 years, average age of normal control population was 72.3 years  Education: Mean number of years spent in formal education of MCI population was 15.8 years, and for normal control population was 16.5 years.  Gender: 48% of MCI population was female, while 60% of normal control population was female.  Ethnicity: 78% of MCI population and 83% of normal control group was white. | Risks vs benefit assessment  Consent and ethics |  |
| (39) | Ashford (2021) | USA | Case Report / Series | Primary Quantitative Research: Reporting of study design changes made to improve inclusivity of AD clinical trial recruitment and resulting changes observed. | Alzheimer's | Ethnicity: Enrolment numbers for ethnoculturally diverse (i.e. not white) were recorded and showed a 25% increase since implementation of changes. |  | Protocol design and management |
| (40) | Jimoh (2021) | USA | Systematic review | Secondary research: Systematic review and narrative synthesis to investigate the application of Mental Capacity Act (2005) and associated provisions to improve research participation for those with capacity and communication difficulties from 2007 to 2019. | Persons lacking capacity to consent | N/A | Consent and ethics | Consent and ethics |
| (41) | Pittman (2021) | USA | Systematic review | Secondary research: Systematic review of hearing loss management studies with focus on ethnic and gender representation in their cohorts. | Bilateral sensorineural hearing loss | Ethnicity: Among 125 clinical studies regarding hearing loss management, only 16 (12.8%) reported race/ethnicity. A mean of 30% (range, 1.96%-100%) of participants were from racial or ethnic minority groups among the 16 studies that reported race/ethnicity.  Gender: Among 125 clinical studies regarding hearing loss management, 88 (70.4%) reported sex. A mean of 41% (range, 1.55%-77.5%) of participants were female among studies that reported sex | Protocol design and management |  |
| (42) | McKinney (2021) | UK | Case Report / Series | Short Communication - Researcher Perspective: Reflections from a research team on experience of recruiting for and running clinical trial for children with profound autism. | Non-verbal, minimally verbal or those with intellectual disability | Age: All participants in the trial being reflected on are children. | Protocol design and management  Risks vs benefit assessment  Consent and ethics  Systemic and logistical factors | Protocol design and management |
| (43) | Langbaum (2020) | Switzerland | Case Report / Series | Primary Quantitative Research: Statistical analysis of results from application of a new recruitment registry for people with AD. | Mild Alzheimer's | Age: Members had a mean age of 63.3 (SD 11.7) years  Gender: Members were predominately women (75%)  Ethnicity: of those who provided race, 76% were white.  Family History: 50% reported a family history of AD or other dementia | Internal vs external validity | Systemic and logistical factors |
| (44) | Hsiao (2019) | UK | Commentary/Opinion/Editorial | Short Communication - Researcher Perspective: Commentary piece giving consolidated and coordinated advice on the best practices for clinical care and clinical research for individuals who suffer from FOP, as established by the the best practices for clinical care and clinical research for individuals who suffer from FOP | fibrodysplasia ossificans progressiva (FOP). | N/A | Protocol design and management  Risks vs benefit assessment  Systemic and logistical factors | Protocol design and management |
| (45) | Peckham (2018) | UK | Case Report / Series | Short Communication - Researcher Perspective: Reflections from a research team on experience of recruiting for and running clinical trial for a clinical trial on smoking cessation for people with severe mental ill health. | Serious Mental Health | N/A | Protocol design and management | Protocol design and management |
| (46) | Bardach (2017) | USA | Quantitative Study | Primary Qualitative Research - Patient and Carer Perspective: Survey study with quantitative questions about importance of different research motivators. Completed by individuals engaged in AD treatment and prevention Clinical trials (n=87), including participants (n=68) and study partners (n=16). | Alzheimer's | Age: Respondents had a mean age of 72.4 years (range of 54.6–89.8)  Ethnicity: 87.4% of respondents were Caucasian.  Gender: Just over half of respondents were male (55.2%). | Risks vs benefits assessment  Systemic and logistical factors | Risks vs benefits assessment |
| (47) | Wade (2017) | USA | Commentary/Opinion/Editorial | Short Communication - Researcher Perspective: Commentary written by researchers with experience implementing eight RCTs of family-centered interventions to reduce child behavior problems and caregiver/parent distress | Traumatic Brain Injury | N/A |  | Protocol design and management |
| (48) | Siddiqui (2015) | USA | Case Report / Series | Primary Quantitative Research: Statistical analysis of data from a clinical trial about cardiovascular disease among people with serious mental illness to examine racial differences in interest, enrolment, and potential barriers to participation. | Serious mental illness | Ethnicity: Key differences were analysed between the two major ethnic groups who took part in the trial – African Americans and Caucasians. | Systemic and logistical factors  Protocol design and management | Protocol design and management  Systemic and logistical factors |
| (49) | Lipsman (2012) | UK | Commentary/Opinion/Editorial | Short Communication - Researcher Perspective: Commentary piece specifically discussing challenge of gaining informed consent in trial recruitment process from populations with psychiatric disease. | Psychiatric disorders | N/A | Protocol design and management  Risks vs benefit assessment  Consent and ethics | Risks vs benefits assessment |
| (50) | Schwartz (1995) | UK | Quantitative analysis | Primary Quantitative Research: Statistical analysis of sociodemographic and medical characteristics as predictors of participation from participants and non-participants in a clinical trial for people with multiple sclerosis. | Multiple Sclerosis | Age: The mean age of the sample of 325 was 46.7 years (SD = 11.0)  Marital Status: 70% of cohort were married.  Gender: Women made up 66% of the cohort.  Employment status: 29% of people in the cohort were in active, 5% were disabled from work employment.  Income: Median family income of 56% of cohort was less than $39,000 | Risks vs benefits  Consent and ethics | Protocol design and management  Risks vs benefits assessment |
| (51) | Fisher (2021) | Canada | Case Report / Series | Short Communication - Researcher Perspective: Reflections from a research team on experience of recruiting for and running a clinical trial for people with depression and traumatic brain injury via digital solutions. | Depression and TBI | N/A | Risks vs benefits  Systemic and logistical factors | Protocol design and management  Consent and ethics |
| (52) | Reuter (2021) | Canada | Quantitative Study | Primary Quantitative Research: Statistical analysis of recruitment data to compare the effectiveness of general practice (traditional) and digital (online) methods of recruiting stroke survivors to a clinical mobility study. | Stroke | N/A |  | Protocol design and management |
| (53) | Brintnall-Karabelas (2011) | USA | Qualitative study | Primary Qualitative Research - Patient Perspective: Study analysing reasons for non-participation of eligible people with mental illness recorded in the National Institute of Mental Health (NIMH) database. | Mental Illness | N/A | Risks vs benefits  Systemic and logistical factors | Protocol design and management  Risks vs benefits assessment |
| (54) | Lennox (2005) | Australia | Mixed-methods study | Primary Quantitative Research and Primary Qualitative Research – Patient Perspective: Analysis of recruitment outcomes on adoption of new strategies for recruitment in an ongoing clinical trial for people with intellectual disability. Additionally, qualitative interviews in focus groups were conducted with participants recruited about their thoughts about the recruitment process (n=265) | Intellectual Disability | Organisational affiliation: 36% (n = 103) were recruited from 26 large government organizations, 22% (n = 62) from 15 large non-government organizations; 15% (n = 44) from 49 small non-government organizations; 12% (n = 35) from 15 church organizations; 5% (n = 15) from six private hostels, and 10% (n = 28) from unidentified sources. | Risks vs benefits  Systemic and logistical factors  Consent and ethics | Protocol design and management  Systemic and logistical factors |
| (55) | Barron (2004) | USA | Commentary/Opinion/Editorial | Short Communication - Researcher Perspective: Discussion by researchers with background in geriatric medicine about informed consent processes as a barrier to recruitment for frail older people. | Frailty | N/A | Risks vs benefits  Systemic and logistical factors  Consent and ethics | Consent and ethics |
| (56) | Jongsma (2016) | Netherlands | Literature Review | Secondary Research: Literature review of dementia research and its exclusion criteria in order to get a clearer picture whether the research participants represent the general dementia population. Discussion of exclusion criteria as a barrier to fair recruitment. | Dementia | N/A | Internal vs external validity | Protocol design and management  Internal vs external validity |

1. Brooks SP, Bubela T. Application of protection motivation theory to clinical trial enrolment for pediatric chronic conditions. *BMC Pediatrics*. 2020;20(1). https://doi.org/10.1186/S12887-020-2014-5/FIGURES/3.

2. Witham MD. Bridging the gap between the laboratory and the clinic for patients with sarcopenia. *Biogerontology*. 2019;20(2): 241–248. https://doi.org/10.1007/S10522-018-09793-Z/FIGURES/1.

3. Park L, Kouhanim C, Lee S, Mendoza Z, Patrick K, Gertsik L, et al. Implementing a Memory Clinic Model to Facilitate Recruitment into Early Phase Clinical Trials for Mild Cognitive Impairment and Alzheimer’s Disease. *The journal of prevention of Alzheimer’s disease*. 2019;6(2): 135–138. https://doi.org/10.14283/JPAD.2019.8/TABLES/1.

4. Bayer A, Fish M. The Doctor’s Duty to the Elderly Patient in Clinical Trials. *Drugs and Aging*. 2003;20(15): 1087–1097. https://doi.org/10.2165/00002512-200320150-00002/FIGURES/TAB1.

5. Humphreys K, Blodgett JC, Roberts LW. The exclusion of people with psychiatric disorders from medical research. *Journal of Psychiatric Research*. 2015;70: 28–32. https://doi.org/10.1016/J.JPSYCHIRES.2015.08.005.

6. Fisher CB, Pearson JL, Kim S, Reynolds CF. Ethical issues in including suicidal individuals in clinical research. *IRB*. 2002;24(5): 9–14. https://doi.org/10.2307/3563804.

7. Carey MP, Morrison-Beedy D, Carey KB, Maisto SA, Gordon CM, Pedlow CT. Psychiatric Outpatients Report Their Experiences as Participants in a Randomized Clinical Trial. *The Journal of nervous and mental disease*. 2001;189(5): 299. https://doi.org/10.1097/00005053-200105000-00005.

8. Jensen JS, Reiter-Theil S, Celio DA, Jakob M, Vach W, Saxer FJ. Handling of informed consent and patient inclusion in research with geriatric trauma patients - a matter of protection or disrespect? *Clinical Interventions in Aging*. 2019;14: 321–334. https://doi.org/10.2147/CIA.S191751.

9. Trivedi RB, Humphreys K. Participant exclusion criteria in treatment research on neurological disorders: Are unrepresentative study samples problematic? *Contemporary clinical trials*. 2015;44: 20–25. https://doi.org/10.1016/J.CCT.2015.07.009.

10. Schneider LS, Olin JT, Lyness SA, Chui HC. Eligibility of Alzheimer’s Disease Clinic Patients for Clinical Trials. *Journal of the American Geriatrics Society*. 1997;45(8): 923–928. https://doi.org/10.1111/J.1532-5415.1997.TB02960.X.

11. Lehoux C, Lefebvre AA, Létourneau K, Viau H, Gosselin D, Szatmari P, et al. A pilot feasibility study of an extension of the acquaintanceship recruitment procedure in recent-onset psychosis. *Journal of Nervous and Mental Disease*. 2005;193(8): 560–563. https://doi.org/10.1097/01.NMD.0000172680.18302.80.

12. Karlawish JHT, Casarett D. Addressing the ethical challenges of clinical trials that involve patients with dementia. *Journal of Geriatric Psychiatry and Neurology*. 2001;14(4): 222–228. https://doi.org/10.1177/089198870101400407.

13. Humphreys K, Weisner C. Use of exclusion criteria in selecting research subjects and its effect on the generalizability of alcohol treatment outcome studies. *American Journal of Psychiatry*. 2000;157(4): 588–594. https://doi.org/10.1176/APPI.AJP.157.4.588/ASSET/IMAGES/LARGE/AY14T1.JPEG.

14. Hammers DB, Foster NL, Hoffman JM, Greene TH, Duff K. Neuropsychological, Psychiatric, and Functional Correlates of Clinical Trial Enrollment. *The journal of prevention of Alzheimer’s disease*. 2019;6(4): 242–247. https://doi.org/10.14283/JPAD.2019.38/TABLES/1.

15. Jones H, Cipriani A. Barriers and incentives to recruitment in mental health clinical trials. *Evidence-Based Mental Health*. 2019;22(2): 49–50. https://doi.org/10.1136/EBMENTAL-2019-300090.

16. Heller JH, Spiridigliozzi GA, Crissman BG, Sullivan-Saarela JA, Li JS, Kishnani PS. Clinical trials in children with Down syndrome: Issues from a cognitive research perspective. *American Journal of Medical Genetics Part C: Seminars in Medical Genetics*. 2006;142C(3): 187–195. https://doi.org/10.1002/AJMG.C.30103.

17. McQueen M. Patient and Physician Attitudes Regarding Clinical Trials in... : Journal of Neuroscience Nursing. *Journal of Neuroscience Nursing*. 2008;40(6). https://journals.lww.com/jnnonline/Abstract/2008/12000/Patient_and_Physician_Attitudes_Regarding_Clinical.5.aspx

18. Lembke A, Humphreys K. A call to include people with mental illness and substance use disorders alongside ‘regular’ smokers in smoking cessation research. *Tobacco Control*. 2016;25(3): 261–262. https://doi.org/10.1136/TOBACCOCONTROL-2014-052215.

19. Schneider RB, Myers TL, Rowbotham HM, Luff MK, Amodeo K, Sharma S, et al. A Virtual Cohort Study of Individuals at Genetic Risk for Parkinson’s Disease: Study Protocol and Design. *Journal of Parkinson’s Disease*. 2020;10(3): 1195. https://doi.org/10.3233/JPD-202019.

20. Fox RJ, Chataway J. Advancing trial design in progressive multiple sclerosis. *Multiple sclerosis (Houndmills, Basingstoke, England)*. 2017;23(12): 1573–1578. https://doi.org/10.1177/1352458517729768.

21. Diver C. *Recruitment and participation in the stem cell trial of recovery enhancement-3 (stems-3): experiences of stroke survivors and their carer’s | Cochrane Library*. Cochrane Central Register of Controlled Trials. https://www.cochranelibrary.com/central/doi/10.1002/central/CN-01470661/full [Accessed 7th May 2022].

22. Shapiro ET, Schamel JT, Parker KA, Randall LA, Frew PM. The role of functional, social, and mobility dynamics in facilitating older African Americans participation in clinical research. *Open Access Journal of Clinical Trials*. 2017;9: 21–30. https://doi.org/10.2147/OAJCT.S122422.

23. Helmchen H. Problems of informed consent for clinical trials in psychiatry. *Controlled clinical trials*. 1981;1(4): 435–440. https://doi.org/10.1016/0197-2456(81)90049-0.

24. Rosende-Roca M, Abdelnour C, Esteban E, Tartari JP, Alarcon E, Martínez-Atienza J, et al. The role of sex and gender in the selection of Alzheimer patients for clinical trial pre-screening. *Alzheimer’s Research & Therapy*. 2021;13(1). https://doi.org/10.1186/S13195-021-00833-4.

25. Wójcik D, Szczechowiak K, Zboch M, Pikala M. Effectiveness of the Open Screening Programs in Recruiting Subjects to Prodromal and Mild Alzheimer’s Disease Clinical Trials. *Journal of Prevention of Alzheimer’s Disease*. 2020;7(4): 251–255. https://doi.org/10.14283/JPAD.2020.15/FIGURES/2.

26. Studenski S. Target populations for clinical trials. *The Journal of Nutrition, Health & Aging*. 2009;13.

27. Witham MD, Anderson E, Carroll CB, Dark PM, Down K, Hall AS, et al. Ensuring that COVID-19 research is inclusive: guidance from the NIHR INCLUDE project. *BMJ Open*. 2020;10(11): e043634. https://doi.org/10.1136/BMJOPEN-2020-043634.

28. Withall J, Group for the RSR, Greaves CJ, Group for the RSR, Thompson JL, Group for the RSR, et al. The Tribulations of Trials: Lessons Learnt Recruiting 777 Older Adults Into REtirement in ACTion (REACT), a Trial of a Community, Group-Based Active Aging Intervention Targeting Mobility Disability. *The Journals of Gerontology: Series A*. 2020;75(12): 2387–2395. https://doi.org/10.1093/GERONA/GLAA051.

29. Kannisto KA, Korhonen J, Adams CE, Koivunen MH, Vahlberg T, Välimäki MA. Factors Associated With Dropout During Recruitment and Follow-Up Periods of a mHealth-Based Randomized Controlled Trial for Mobile.Net to Encourage Treatment Adherence for People With Serious Mental Health Problems. *J Med Internet Res 2017;19(2):e46 https://www.jmir.org/2017/2/e46*. 2017;19(2): e6417. https://doi.org/10.2196/JMIR.6417.

30. Emre M, Ford PJ, Bilgiç B, Uç EY. Cognitive impairment and dementia in Parkinson’s disease: Practical issues and management. *Movement Disorders*. 2014;29(5): 663–672. https://doi.org/10.1002/MDS.25870.

31. van Rookhuijzen AE, Touwen DP, de Ruijter W, Engberts DP, van der Mast RC. Deliberating clinical research with cognitively impaired older people and their relatives: an ethical add-on study to the protocol “Effects of Temporary Discontinuation of Antihypertensive Treatment in the Elderly (DANTE) with Cognitive Impairment.” *The American journal of geriatric psychiatry : official journal of the American Association for Geriatric Psychiatry*. 2014;22(11): 1233–1240. https://doi.org/10.1016/J.JAGP.2013.04.005.

32. Kogan JN, Bauer MS, Dennehy EB, Miklowitz DJ, Gonzalez JM, Thompson PM, et al. Increasing minority research participation through collaboration with community outpatient clinics: the STEP-BD Community Partners Experience. 2009; https://doi.org/10.1177/1740774509338427.

33. Veenstra MY, Walsh PN, van Schrojenstein Lantman-De Valk HMJ, Haveman MJ, Linehan C, Kerr MP, et al. Sampling and ethical issues in a multicenter study on health of people with intellectual disabilities. *Journal of Clinical Epidemiology*. 2010;63(10): 1091–1100. https://doi.org/10.1016/J.JCLINEPI.2009.12.001.

34. Africano-Oliver P, Dickens S, Ahmed Z, Bouras N, Cooray S, Deb S, et al. Overcoming the barriers experienced in conducting a medication trial in adults with aggressive challenging behaviour and intellectual disabilities. *Journal of Intellectual Disability Research*. 2010;54(1): 17–25. https://doi.org/10.1111/J.1365-2788.2009.01195.X.

35. House SA, Shubkin CD, Lahey T, Brosco JP, Lantos J. COVID-19 trial enrollment for those who cannot consent: Ethical challenges posed by a pandemic. *Pediatrics*. 2020;146(5). https://doi.org/10.1542/PEDS.2020-010728/75340.

36. Grill JD, Karlawish J. Addressing the challenges to successful recruitment and retention in Alzheimer’s disease clinical trials. *Alzheimer’s Research and Therapy*. 2010;2(6): 1–11. https://doi.org/10.1186/ALZRT58/TABLES/3.

37. Ridda I, Lindley R, MacIntyre RC. The challenges of clinical trials in the exclusion zone: The case of the frail elderly. *Australasian Journal on Ageing*. 2008;27(2): 61–66. https://doi.org/10.1111/J.1741-6612.2008.00288.X.

38. Jefferson AL, Lambe S, Moser DJ, Byerly LK, Ozonoff A, Karlawish JH. Decisional Capacity for Research Participation in Individuals with Mild Cognitive Impairment. *Journal of the American Geriatrics Society*. 2008;56(7): 1236–1243. https://doi.org/10.1111/J.1532-5415.2008.01752.X.

39. Ashford MT, Ozioma O. EFFORTS TO IMPROVE RECRUITMENT AND ENGAGEMENT OF UNDERREPRESENTED POPULATIONS IN THE ALZHEIMER’S DISEASE NEUROIMAGING INITIATIVE (ADNI) STUDY. *The Journal of Prevention of Alzheimer’s Disease* . 2021;8(1): S1–S72. https://doi.org/10.14283/JPAD.2021.57.

40. Jimoh OF, Ryan H, Killett A, Shiggins C, Langdon PE, Heywood R, et al. A systematic review and narrative synthesis of the research provisions under the Mental Capacity Act (2005) in England and Wales: Recruitment of adults with capacity and communication difficulties. *PLOS ONE*. 2021;16(9): e0256697. https://doi.org/10.1371/JOURNAL.PONE.0256697.

41. Pittman CA, Roura R, Price C, Lin FR, Marrone N, Nieman CL. Racial/Ethnic and Sex Representation in US-Based Clinical Trials of Hearing Loss Management in Adults: A Systematic Review. *JAMA Otolaryngology–Head & Neck Surgery*. 2021;147(7): 656–662. https://doi.org/10.1001/JAMAOTO.2021.0550.

42. McKinney A, Weisblatt EJL, Hotson KL, Bilal Ahmed Z, Dias C, BenShalom D, et al. Overcoming hurdles to intervention studies with autistic children with profound communication difficulties and their families. *Autism*. 2021;25(6): 1627–1639. https://doi.org/10.1177/1362361321998916.

43. Langbaum JB, High N, Nichols J, Kettenhoven C, Reiman EM, Tariot PN. The Alzheimer’s Prevention Registry: A Large Internet-Based Participant Recruitment Registry to Accelerate Referrals to Alzheimer’s-Focused Studies. *Journal of Prevention of Alzheimer’s Disease*. 2020;7(4): 242–250. https://doi.org/10.14283/JPAD.2020.31/TABLES/1.

44. Hsiao EC, di Rocco M, Cali A, Zasloff M, al Mukaddam M, Pignolo RJ, et al. Special considerations for clinical trials in fibrodysplasia ossificans progressiva (FOP). *British Journal of Clinical Pharmacology*. 2019;85(6): 1199–1207. https://doi.org/10.1111/BCP.13777.

45. Peckham E, Arundel C, Bailey D, Callen T, Cusack C, Crosland S, et al. Successful recruitment to trials: Findings from the SCIMITAR+ Trial. *Trials*. 2018;19(1): 1–6. https://doi.org/10.1186/S13063-018-2460-7/FIGURES/1.

46. Bardach SH, Holmes SD, Jicha GA. Motivators for Alzheimer’s disease clinical trial participation. *Aging Clinical and Experimental Research*. 2018;30(2): 209–212. https://doi.org/10.1007/S40520-017-0771-2/FIGURES/2.

47. Wade SL, Kurowski BG. Behavioral Clinical Trials in Moderate to Severe Pediatric Traumatic Brain Injury: Challenges, Potential Solutions, and Lessons Learned. *The Journal of head trauma rehabilitation*. 2017;32(6): 433. https://doi.org/10.1097/HTR.0000000000000323.

48. Siddiqui M, Cooper LA, Appel LJ, Yu A, Charleston J, Gennusa J, et al. Recruitment and Enrollment of African Americans and Caucasians in a Health Promotion Trial for Persons with Serious Mental Illness. *Ethnicity & disease*. 2015;25(1): 72. /pmc/articles/PMC4663046/

49. Lipsman N, Giacobbe P, Bernstein M, Lozano AM. Informed consent for clinical trials of deep brain stimulation in psychiatric disease: challenges and implications for trial design. *Journal of Medical Ethics*. 2012;38(2): 107–111. https://doi.org/10.1136/JME.2010.042002.

50. Schwartz CE, Fox BH. Who says yes? Identifying selection biases in a psychosocial intervention study of multiple sclerosis. *Social Science & Medicine*. 1995;40(3): 359–370. https://doi.org/10.1016/0277-9536(94)E0092-7.

51. Fisher LB, Tuchman S, Curreri AJ, Markgraf M, Nyer MB, Cassano P, et al. Transitioning From In-Person to Remote Clinical Research on Depression and Traumatic Brain Injury During the COVID-19 Pandemic: Study Modifications and Preliminary Feasibility From a Randomized Controlled Pilot Study. *JMIR Form Res 2021;5(12):e28734 https://formative.jmir.org/2021/12/e28734*. 2021;5(12): e28734. https://doi.org/10.2196/28734.

52. Reuter K, Liu C, Le NQ, Angyan P, Finley JM. General Practice and Digital Methods to Recruit Stroke Survivors to a Clinical Mobility Study: Comparative Analysis. *J Med Internet Res 2021;23(10):e28923 https://www.jmir.org/2021/10/e28923*. 2021;23(10): e28923. https://doi.org/10.2196/28923.

53. Brintnall-Karabelas J, Sung S, Cadman ME, Squires C, Whorton K, Pao M. Improving recruitment in clinical trials: Why eligible participants decline. *Journal of Empirical Research on Human Research Ethics*. 2011;6(1): 69–74. https://doi.org/10.1525/jer.2011.6.1.69.

54. Lennox N, Taylor M, Rey-Conde T, Bain C, Purdie DM, Boyle F. Beating the barriers: recruitment of people with intellectual disability to participate in research. *Journal of intellectual disability research : JIDR*. 2005;49(Pt 4): 296–305. https://doi.org/10.1111/J.1365-2788.2005.00618.X.

55. Barron JS, Duffey PL, Jo Byrd L, Campbell R, Ferrucci L. Informed consent for research participation in frail older persons. *Aging Clinical and Experimental Research 2004 16:1*. 2013;16(1): 79–85. https://doi.org/10.1007/BF03324536.

56. Jongsma KR, van Bruchem-Visser RL, van de Vathorst S, Raso FUSM. Has dementia research lost its sense of reality? A descriptive analysis of eligibility criteria of Dutch dementia research protocols.
